# Supplementary material for: Postinspiratory complex acts as a gating mechanism regulating swallow-breathing coordination and other laryngeal behaviors
Source: bioRxiv. 2023 Jan 20:2023.01.18.524513. Preprint. [Version 1] doi: 10.1101/2023.01.18.524513 (PMC9882227; doi:10.1101/2023.01.18.524513)
Supplement: Supplement 1 [file NIHPP2023.01.18.524513v1-supplement-1.pdf]

bioRxiv preprint doi: https://doi.org/10.1101/2023.01.18.524513; this version posted January 20, 2023. The copyright holder for this preprint (which was not certified by peer review) is the author/funder, who has granted bioRxiv a license to display the preprint in perpetuity. It is made available under aCC-BY 4.0 International license.

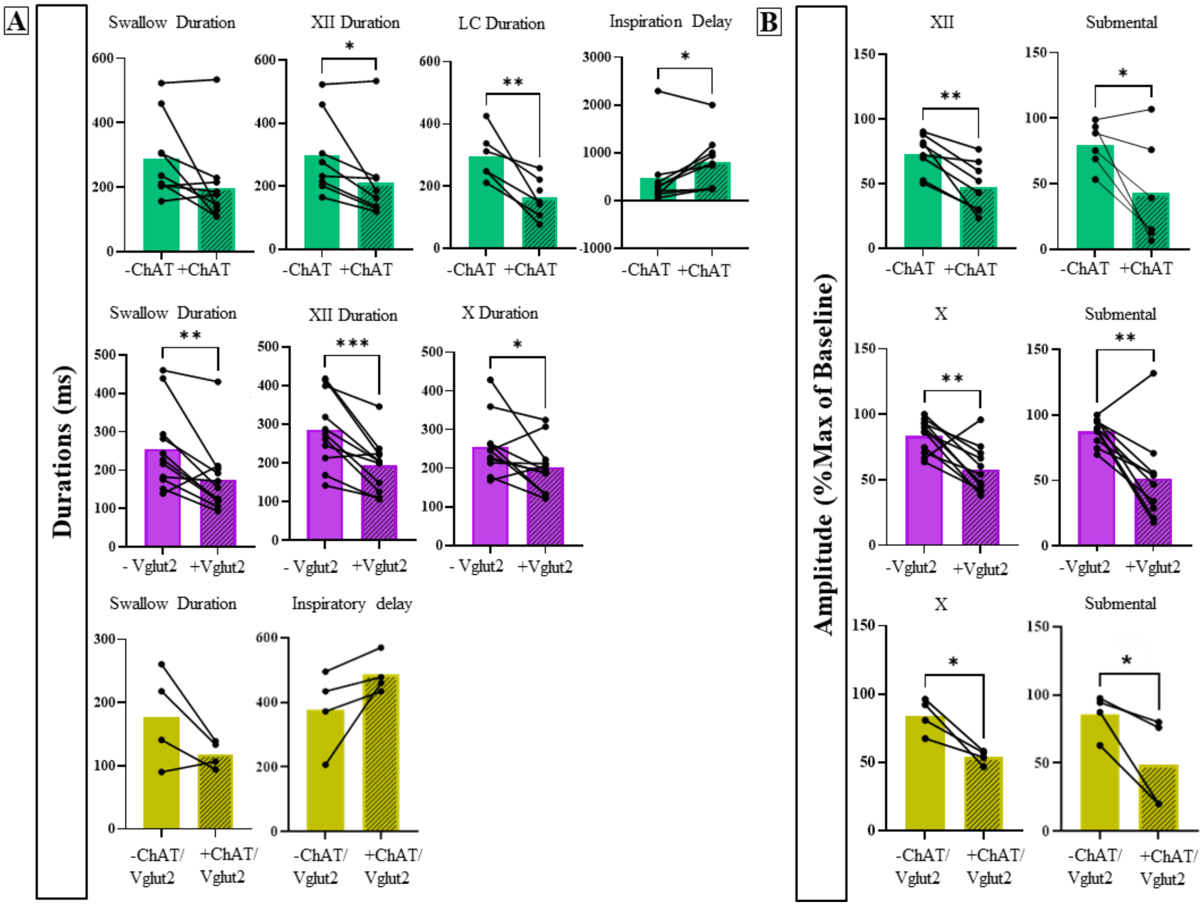


**Supplemental Figure 1.** PiCo triggered swallows have a decrease in duration and amplitude compared to water triggered swallows. A) Comparison of durations and B) amplitude in swallow-related characteristics for water swallows (-) and PiCo stimulated swallows (+) in ChAT (green, N=10), Vglut2 (purple, N=11) and ChAT/Vglut2 (gold, N=4). Abbreviations: X, vagus nerve; XII, hypoglossal nerve; LC, laryngeal complex.

bioRxiv preprint doi: https://doi.org/10.1101/2023.01.18.524513; this version posted January 20, 2023. The copyright holder for this preprint (which was not certified by peer review) is the author/funder, who has granted bioRxiv a license to display the preprint in perpetuity. It is made available under aCC-BY 4.0 International license.

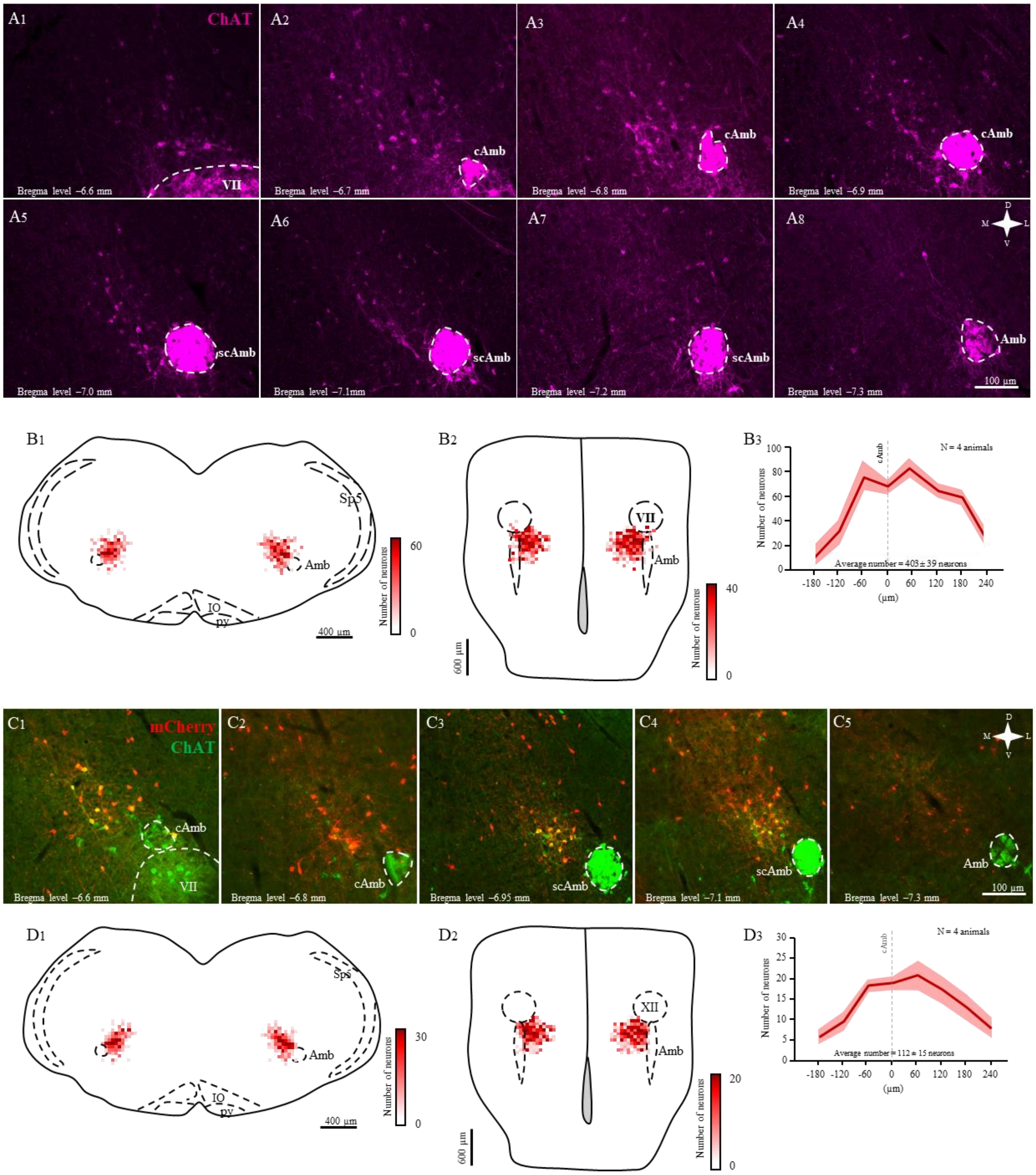


**Supplemental Figure 2.** Anatomical characterization of PiCo region. A) Coronal views (Bregma level -6.6 to -7.3 mm) of the ventro-medial medulla showing the location of the ChAT neurons (magenta) in PiCo region. B) Heat map showing the density of ChAT immunoreactive neurons from 1) coronal and 2) ventral view of 4 animals. B3) Rostro-caudal distribution of the total number of ChAT immunoreactive counted 1:2 series of 25 µm sections into PiCo. C) Coronal views (Bregma level -6.6 to -7.3 mm) of the ventro-medial medulla showing the location of the double conditioned ChAT/Vglut2/Ai65 neurons (red) in PiCo region. D) Heat map showing the density of ChAT/Vglut2/Ai65 neurons from 1) coronal and 2) ventral view of 4 animals. D3)

bioRxiv preprint doi: https://doi.org/10.1101/2023.01.18.524513; this version posted January 20, 2023. The copyright holder for this preprint (which was not certified by peer review) is the author/funder, who has granted bioRxiv a license to display the preprint in perpetuity. It is made available under aCC-BY 4.0 International license.

Rostro-caudal distribution of the total number of ChAT/Vglut2/Ai65 neurons counted 1:2 series of 25 µm sections into PiCo. Abbreviations: cAmb, nucleus ambiguus pars compacta; scAmb, nucleus ambiguus pars semi-compacta; Amb, nucleus ambiguus pars non-compacta; VII, facial motor nucleus; IO, inferior olive; py, pyramidal tract; Sp5, spinal trigeminal nucleus; VII, facial motor nucleus.

bioRxiv preprint doi: https://doi.org/10.1101/2023.01.18.524513; this version posted January 20, 2023. The copyright holder for this preprint (which was not certified by peer review) is the author/funder, who has granted bioRxiv a license to display the preprint in perpetuity. It is made available under aCC-BY 4.0 International license.

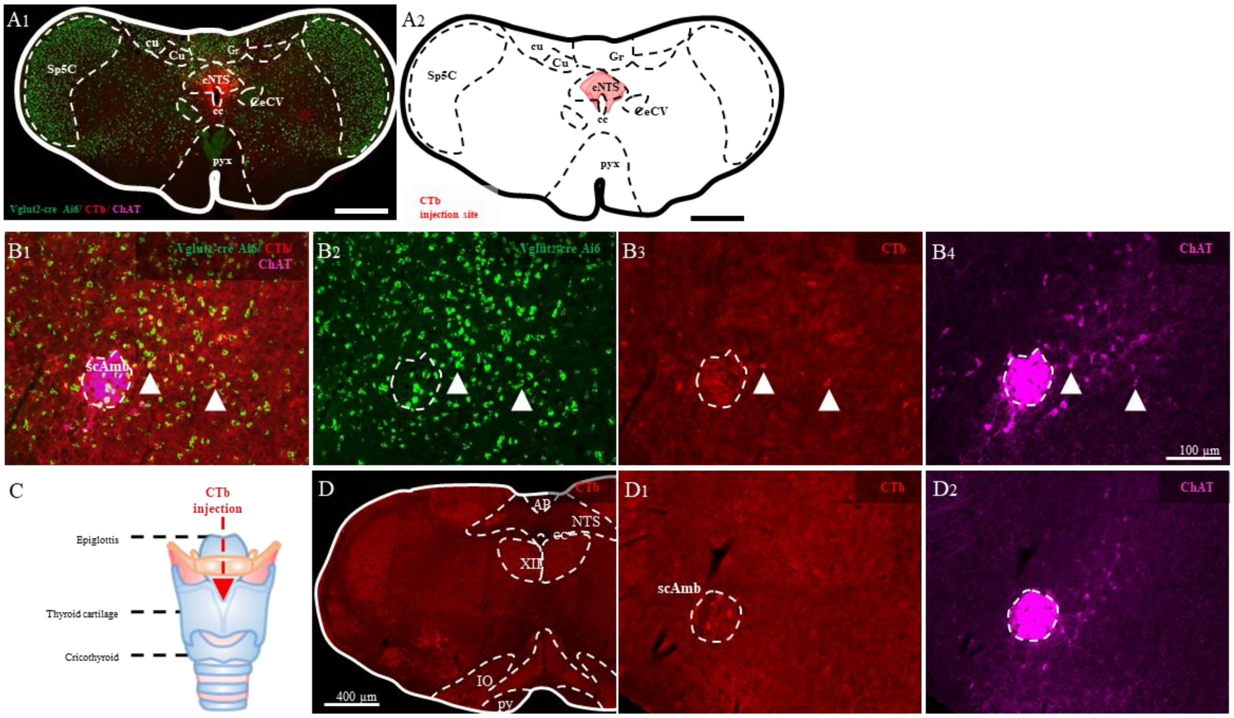


**Supplemental Figure 3.** PiCo cells retrogradely labelled with CTb following deposit into cNTS. A) Photomicrography and schematic drawing of retrograde tracer CTb injected into cNTS. B) Coronal views (Bregma level -7.1 mm) of PiCo showing the location of the retrogradely stained cells with CTb (red), ChAT (magenta) and Vglut2 (green) neurons in PiCo region. White arrows indicate some examples of triple retrogradely labelled cells in PiCo. C) Drawing of the larynx where CTb has been deposited. D) Coronal views (Bregma level -7.1 mm) of PiCo showing no CTb (red) stained neurons after CTb injection into the larynx. Abbreviations: scAmb, nucleus ambiguus pars semi-compacta; Sp5C, caudal part of spinal trigeminal nucleus; Cu, cuneate nucleus; cu, cuneate fasciculus; AP, area postrema; Gr, gracile nucleus; cc, central canal; CeCV, central cervical nucleus; cNTS, caudal nucleus of solitary tract; IO, inferior olive; py, pyramidal tract; pyx, pyramidal decussation; XII, hypoglossal nucleus.

bioRxiv preprint doi: https://doi.org/10.1101/2023.01.18.524513; this version posted January 20, 2023. The copyright holder for this preprint (which was not certified by peer review) is the author/funder, who has granted bioRxiv a license to display the preprint in perpetuity. It is made available under aCC-BY 4.0 International license.

**Table S1.** Provides means and standard deviations (SD) for vagus and laryngeal complex duration during laryngeal activation in response to increasing stimuli in ChAT, Vglut2, and ChAT/Vglut2 mice.

| Laryngeal Activation | + ChAT Stimulation mean ( SD ) | + Vglut2 Stimulation mean ( SD ) | + ChAT/Vglut2 Stimulation mean ( SD ) |
|---|---|---|---|
| **40ms** | | | |
| Vagus Duration (ms) | 77 ( 14 ) | 85 ( 8 ) | 75 ( 11 ) |
| Laryngeal Complex Duration (ms) | 76 ( 9 ) | 81 ( 13 ) | 72 ( 7 ) |
| **80ms** | | | |
| Vagus Duration (ms) | 108 ( 16 ) | 101 ( 27 ) | 106 ( 12 ) |
| Laryngeal Complex Duration (ms) | 105 ( 16 ) | 103 ( 23 ) | 119 ( 14 ) |
| **120ms** | | | |
| Vagus Duration (ms) | 140 ( 31 ) | 123 ( 55 ) | 142 ( 23 ) |
| Laryngeal Complex Duration (ms) | 131 ( 20 ) | 125 ( 57 ) | 151 ( 6 ) |
| **160ms** | | | |
| Vagus Duration (ms) | 163 ( 25 ) | 114 ( 62 ) | 142 ( 42 ) |
| Laryngeal Complex Duration (ms) | 164 ( 32 ) | 128 ( 68 ) | 175 ( 19 ) |
| **200ms** | | | |
| Vagus Duration (ms) | 197 ( 62 ) | 132 ( 50 ) | 165 ( 61 ) |
| Laryngeal Complex Duration (ms) | 243 ( 146 ) | 152 ( 57 ) | 208 ( 25 ) |
| **Average** | | | |
| Vagus Duration (ms) | 145 ( 33 ) | 100 ( 24 ) | 127 ( 30 ) |
| Laryngeal Complex Duration (ms) | 133 ( 40 ) | 103 ( 28 ) | 149 ( 13 ) |

bioRxiv preprint doi: https://doi.org/10.1101/2023.01.18.524513; this version posted January 20, 2023. The copyright holder for this preprint (which was not certified by peer review) is the author/funder, who has granted bioRxiv a license to display the preprint in perpetuity. It is made available under aCC-BY 4.0 International license.

**Table S2.** Provides means, standard deviations (SD), *p*-values and the direction of change for swallow related parameters when evoked by water (water swallows) and optogenetic stimulation of PiCo in A) ChAT, B) Vglut2 and C) ChAT/Vglut2 mice.

| **A** | **Water Swallow** | | **+ ChAT Stimulation** | | | |
|---|---|---|---|---|---|---|
| | **mean** | **( SD )** | **mean** | **( SD )** | ***p*-value** | **Change** |
| **(*n*=10)** | | | | | | |
| Swallow Duration (ms) | 290 | ( 125 ) | 198 | ( 125 ) | 0.06 | - |
| Hypoglossal Duration (ms) | 297 | ( 129 ) | 212 | ( 127 ) | 0.03 | ↓ |
| Vagus Duration (ms) | 294 | ( 101 ) | 224 | ( 112 ) | 0.05 | ↓ |
| Laryngeal Complex Duration (ms) | 297 | ( 78 ) | 163 | ( 63 ) | 0.009 | ↓ |
| *Schluckatmung* Duration (ms) | 228 | ( - ) | - | ( - ) | - | - |
| Diaphragm Inter-Burst Interval (ms) | 1002 | ( 691 ) | 1361 | ( 938 ) | 0.06 | - |
| SR Inspiratory Delay (ms) | 482 | ( 696 ) | 811 | ( 534 ) | 0.04 | ↑ |
| Swallow Sequence (ms) | 24 | ( 40 ) | 18 | ( 19 ) | 0.29 | - |
| Swallow Onset (ms) | 268 | ( 133 ) | 433 | ( 472 ) | 0.24 | - |
| Hypoglossal Amplitude (% max) | 73 | ( 15 ) | 48 | ( 19 ) | 0.002 | ↓ |
| Vagus Amplitude (% max) | 73 | ( 17 ) | 53 | ( 27 ) | 0.06 | - |
| Submental Complex Amplitude (% max) | 78 | ( 17 ) | 43 | ( 40 ) | 0.04 | ↓ |
| Laryngeal Complex Amplitude (% max) | 74 | ( 23 ) | 55 | ( 36 ) | 0.07 | - |
| *Schluckatmung* Amplitude (% max) | - | ( - ) | - | ( - ) | - | - |

| **B** | **Water Swallow** | | **+ Vglut2 Stimulation** | | | |
|---|---|---|---|---|---|---|
| | **mean** | **( SD )** | **mean** | **( SD )** | ***p*-value** | **Change** |
| **(*n*=11)** | | | | | | |
| Swallow Duration (ms) | 256 | ( 108 ) | 175 | ( 94 ) | **0.007** | ↓ |
| Hypoglossal Duration (ms) | 286 | ( 95 ) | 193 | ( 69 ) | **0.001** | ↓ |
| Vagus Duration (ms) | 256 | ( 76 ) | 203 | ( 66 ) | **0.04** | ↓ |
| Laryngeal Complex Duration (ms) | 311 | ( 149 ) | 250 | ( 100 ) | 0.20 | - |
| *Schluckatmung* Duration (ms) | 150 | ( 48 ) | 176 | ( 70 ) | 0.83 | - |
| Diaphragm Inter-Burst Interval (ms) | 834 | ( 488 ) | 749 | ( 316 ) | 0.54 | - |
| SR Inspiratory Delay (ms) | 437 | ( 438 ) | 381 | ( 232 ) | 0.68 | - |
| Swallow Sequence (ms) | 29 | ( 38 ) | 22 | ( 30 ) | 0.37 | - |
| Swallow Onset (ms) | 160 | ( 103 ) | 200 | ( 119 ) | 0.40 | - |
| Hypoglossal Amplitude (% max) | 83 | ( 13 ) | 77 | ( 30 ) | 0.38 | - |
| Vagus Amplitude (% max) | 84 | ( 13 ) | 58 | ( 18 ) | **0.003** | ↓ |
| Submental Complex Amplitude (% max) | 88 | ( 10 ) | 52 | ( 33 ) | **0.004** | ↓ |
| Laryngeal ComplexAmplitude (% max) | 78 | ( 16 ) | 85 | ( 76 ) | 0.76 | - |
| *Schluckatmung* Amplitude (% max) | 80 | ( 12 ) | 170 | ( 152 ) | 0.62 | - |

| **C** | **Water Swallow** | | **+ ChAT/Vglut2 Stimulation** | | | |
|---|---|---|---|---|---|---|
| | **mean** | **( SD )** | **mean** | **( SD )** | ***p*-value** | **Change** |
| **(*n*=4)** | | | | | | |
| Swallow Duration (ms) | 178 | ( 76 ) | 119 | ( 21 ) | 0.14 | - |
| Hypoglossal Duration (ms) | 221 | ( 82 ) | 131 | ( 34 ) | 0.15 | - |
| Vagus Duration (ms) | 233 | ( 86 ) | 162 | ( 31 ) | 0.08 | - |
| Laryngeal Complex Duration (ms) | 251 | ( 83 ) | 230 | ( 138 ) | 0.79 | - |
| *Schluckatmung* Duration (ms) | - | ( - ) | - | ( - ) | - | - |
| Diaphragm Inter-Burst Interval (ms) | 746 | ( 80 ) | 866 | ( 306 ) | 0.37 | - |
| SR Inspiratory Delay (ms) | 377 | ( 124 ) | 486 | ( 59 ) | 0.11 | - |
| Swallow Sequence (ms) | 55 | ( 41 ) | 50 | ( 59 ) | 0.84 | - |
| Swallow Onset (ms) | 185 | ( 53 ) | 231 | ( 142 ) | 0.51 | - |
| Hypoglossal Amplitude (% max) | 85 | ( 12 ) | 72 | ( 25 ) | 0.39 | - |
| Vagus Amplitude (% max) | 84 | ( 13 ) | 54 | ( 5 ) | **0.02** | ↓ |
| Submental Complex Amplitude (% max) | 86 | ( 16 ) | 49 | ( 34 ) | **0.05** | ↓ |
| Laryngeal ComplexAmplitude (% max) | 81 | ( 15 ) | 71 | ( 41 ) | 0.52 | - |
| *Schluckatmung* Amplitude (% max) | - | ( - ) | - | ( - ) | - | - |

bioRxiv preprint doi: https://doi.org/10.1101/2023.01.18.524513; this version posted January 20, 2023. The copyright holder for this preprint (which was not certified by peer review) is the author/funder, who has granted bioRxiv a license to display the preprint in perpetuity. It is made available under aCC-BY 4.0 International license.

**Table S3.** Provides means, standard deviations (SD), *p*-values and the direction of change for swallow related parameters when evoked by water (water swallows) and optogenetic stimulation of PiCo ChAT/Vglut2 mice evoking swallow related motor activity.

| | **Water Swallow** | | **+ ChAT/Vglut2 Stimulation SR Motor Activity** | | | |
|---|---|---|---|---|---|---|
| | **mean** | **( SD )** | **mean** | **( SD )** | ***p*-value** | **Change** |
| **(*n*=3)** | | | | | | |
| Swallow Duration (ms) | 236 | ( 25 ) | 159 | ( 14 ) | **0.03** | ↓ |
| Hypoglossal Duration (ms) | 253 | ( 25 ) | 156 | ( 18 ) | **0.003** | ↓ |
| Vagus Duration (ms) | 247 | ( 33 ) | 165 | ( 20 ) | **0.04** | ↓ |
| Laryngeal Complex Duration (ms) | 256 | ( 2 ) | 168 | ( 30 ) | 0.21 | - |
| *Schluckatmung* Duration (ms) | 182 | ( - ) | - | ( - ) | - | - |
| Diaphragm Inter-Burst Interval (ms) | 684 | ( 74 ) | 744 | ( 318 ) | 0.93 | - |
| SR Inspiratory Delay (ms) | 411 | ( 65 ) | 363 | ( 244 ) | 0.61 | - |
| Swallow Sequence (ms) | 44 | ( 122 ) | -5 | ( 30 ) | 0.79 | - |
| Swallow Onset (ms) | 87 | ( 20 ) | 301 | ( 111 ) | 0.14 | - |
| Hypoglossal Amplitude (% max) | 96 | ( 6 ) | 51 | ( 38 ) | 0.29 | - |
| Vagus Amplitude (% max) | 95 | ( 7 ) | 55 | ( 50 ) | 0.41 | - |
| Submental Complex Amplitude (% max) | 98 | ( 3 ) | 78 | ( 11 ) | 0.18 | - |
| Laryngeal ComplexAmplitude (% max) | 90 | ( 14 ) | 70 | ( 18 ) | 0.53 | - |
| *Schluckatmung* Amplitude (% max) | 81 | ( - ) | - | ( - ) | - | - |

bioRxiv preprint doi: https://doi.org/10.1101/2023.01.18.524513; this version posted January 20, 2023. The copyright holder for this preprint (which was not certified by peer review) is the author/funder, who has granted bioRxiv a license to display the preprint in perpetuity. It is made available under aCC-BY 4.0 International license.

**Table S4.** Provides means, standard deviations (SD), *p*-values and the direction of change for swallow related parameters between male and female mice during water swallows and PiCo stimulated swallows in ChAT mice.

| **Water Swallow** | **Male (*n* =5)** | | **Female (*n* =4)** | | | |
|---|---|---|---|---|---|---|
| | **mean** | **( SD )** | **mean** | **( SD )** | ***p*-value** | **Change** |
| Swallow Duration (ms) | 295 | ( 134 ) | 284 | ( 133 ) | 0.90 | - |
| XII Duration (ms) | 308 | ( 127 ) | 280 | ( 158 ) | 0.79 | - |
| Vagus Duration (ms) | 294 | ( 107 ) | 292 | ( 115 ) | 0.98 | - |
| Laryngeal Complex Duration (ms) | 299 | ( 46 ) | 295 | ( 115 ) | 0.95 | - |
| *Schluckatmung* Duration (ms) | 228 | ( - ) | - | ( - ) | - | - |
| Diaphragm Inter-Burst Interval (ms) | 1261 | ( 835 ) | 678 | ( 304 ) | 0.23 | - |
| SR Inspiratory Delay (ms) | 717 | ( 900 ) | 188 | ( 71 ) | 0.29 | - |
| Swallow Sequence (ms) | 1 | ( 5 ) | 53 | ( 47 ) | **0.04** | ↑ |
| Swallow Onset (ms) | 308 | ( 101 ) | 218 | ( 167 ) | 0.35 | - |
| **+ ChAT Stimulation** | **Male (*n* =6)** | | **Female (*n* =4)** | | | |
| | **mean** | **( SD )** | **mean** | **( SD )** | ***p*-value** | **Change** |
| Swallow Duration (ms) | 239 | ( 150 ) | 137 | ( 36 ) | 0.23 | - |
| XII Duration (ms) | 244 | ( 147 ) | 147 | ( 35 ) | 0.31 | - |
| Vagus Duration (ms) | 262 | ( 121 ) | 150 | ( 33 ) | 0.17 | - |
| Laryngeal Complex Duration (ms) | 204 | ( 46 ) | 109 | ( 32 ) | **0.03** | ↓ |
| *Schluckatmung* Duration (ms) | - | ( - ) | - | ( - ) | - | - |
| Diaphragm Inter-Burst Interval (ms) | 1510 | ( 985 ) | 1138 | ( 954 ) | 0.57 | - |
| SR Inspiratory Delay (ms) | 921 | ( 584 ) | 646 | ( 477 ) | 0.46 | - |
| Swallow Sequence (ms) | 16 | ( 19 ) | 22 | ( 22 ) | 0.64 | - |
| Swallow Onset (ms) | 429 | ( 522 ) | 438 | ( 461 ) | 0.98 | - |

bioRxiv preprint doi: https://doi.org/10.1101/2023.01.18.524513; this version posted January 20, 2023. The copyright holder for this preprint (which was not certified by peer review) is the author/funder, who has granted bioRxiv a license to display the preprint in perpetuity. It is made available under aCC-BY 4.0 International license.

**Table S5.** Provides means, standard deviations (SD), *p*-values and the direction of change for swallow related parameters between male and female mice during water swallows and PiCo stimulated swallows in Vglut2 mice.

| **Water Swallow** | **Male (*n* =7)** | | **Female (*n* =4)** | | | |
|---|---|---|---|---|---|---|
| | **mean** | **( SD )** | **mean** | **( SD )** | ***p*-value** | **Change** |
| Swallow Duration (ms) | 254 | ( 108 ) | 258 | ( 125 ) | 0.95 | - |
| XII Duration (ms) | 295 | ( 92 ) | 269 | ( 112 ) | 0.68 | - |
| Vagus Duration (ms) | 248 | ( 59 ) | 270 | ( 110 ) | 0.68 | - |
| Laryngeal Complex Duration (ms) | 307 | ( 106 ) | 317 | ( 227 ) | 0.92 | - |
| *Schluckatmung* Duration (ms) | 158 | ( 52 ) | 120 | ( - ) | - | - |
| Diaphragm Inter-Burst Interval (ms) | 796 | ( 509 ) | 902 | ( 515 ) | 0.75 | - |
| SR Inspiratory Delay (ms) | 423 | ( 541 ) | 461 | ( 229 ) | 0.90 | - |
| Swallow Sequence (ms) | 30 | ( 46 ) | 29 | ( 23 ) | 0.97 | - |
| Swallow Onset (ms) | 141 | ( 86 ) | 195 | ( 136 ) | 0.43 | - |
| **+ Vglut2 Stimulation** | **Male (*n* =7)** | | **Female (*n* =4)** | | | |
| | **mean** | **( SD )** | **mean** | **( SD )** | ***p*-value** | **Change** |
| Swallow Duration (ms) | 198 | ( 109 ) | 136 | ( 47 ) | 0.32 | - |
| XII Duration (ms) | 207 | ( 79 ) | 170 | ( 50 ) | 0.42 | - |
| Vagus Duration (ms) | 224 | ( 69 ) | 166 | ( 47 ) | 0.17 | - |
| Laryngeal Complex Duration (ms) | 282 | ( 100 ) | 195 | ( 86 ) | 0.18 | - |
| *Schluckatmung* Duration (ms) | 176 | ( 70 ) | - | ( - ) | - | - |
| Diaphragm Inter-Burst Interval (ms) | 645 | ( 207 ) | 932 | ( 422 ) | 0.16 | - |
| SR Inspiratory Delay (ms) | 273 | ( 140 ) | 569 | ( 256 ) | **0.03** | ↑ |
| Swallow Sequence (ms) | 21 | ( 38 ) | 25 | ( 9 ) | 0.88 | - |
| Swallow Onset (ms) | 174 | ( 110 ) | 247 | ( 135 ) | 0.35 | - |

bioRxiv preprint doi: https://doi.org/10.1101/2023.01.18.524513; this version posted January 20, 2023. The copyright holder for this preprint (which was not certified by peer review) is the author/funder, who has granted bioRxiv a license to display the preprint in perpetuity. It is made available under aCC-BY 4.0 International license.

**Table S6.** Provides means, standard deviations (SD), *p*-values and the direction of change for swallow related parameters between male and female mice during water swallows and PiCo stimulated swallows in ChAT/Vglut2 mice.

| **Water Swallow** | **Male (*n* =4)** | | **Female (*n* =1)** | | | |
|---|---|---|---|---|---|---|
| | **mean** | **( SD )** | **mean** | **( SD )** | ***p* -value** | **Change** |
| Swallow Duration (ms) | 219 | ( 55 ) | 140 | ( - ) | 0.29 | - |
| XII Duration (ms) | 254 | ( 63 ) | 204 | ( - ) | 0.52 | - |
| Vagus Duration (ms) | 250 | ( 85 ) | - | ( - ) | - | - |
| Laryngeal Complex Duration (ms) | 267 | ( 73 ) | 189 | ( - ) | 0.41 | - |
| *Schluckatmung* Duration (ms) | - | ( - ) | - | ( - ) | - | - |
| Diaphragm Inter-Burst Interval (ms) | 760 | ( 69 ) | 680 | ( - ) | 0.38 | - |
| SR Inspiratory Delay (ms) | 383 | ( 128 ) | 434 | ( - ) | 0.74 | - |
| Swallow Sequence (ms) | 42 | ( 62 ) | 8 | ( - ) | 0.66 | - |
| Swallow Onset (ms) | 180 | ( 61 ) | 120 | ( - ) | 0.44 | - |
| **+ ChAT/Vglut2 Stimulation** | **Male (*n* =4)** | | **Female (*n* =1)** | | | |
| | **mean** | **( SD )** | **mean** | **( SD )** | ***p* -value** | **Change** |
| Swallow Duration (ms) | 118 | ( 22 ) | 107 | ( - ) | 0.69 | - |
| XII Duration (ms) | 131 | ( 34 ) | 130 | ( - ) | 0.97 | - |
| Vagus Duration (ms) | 161 | ( 32 ) | 145 | ( - ) | 0.67 | - |
| Laryngeal Complex Duration (ms) | 118 | ( 22 ) | 138 | ( - ) | 0.46 | - |
| *Schluckatmung* Duration (ms) | 228 | ( 140 ) | - | ( - ) | - | - |
| Diaphragm Inter-Burst Interval (ms) | 879 | ( 292 ) | 629 | ( - ) | 0.50 | - |
| SR Inspiratory Delay (ms) | 505 | ( 68 ) | 478 | ( - ) | 0.75 | - |
| Swallow Sequence (ms) | 54 | ( 56 ) | 13 | ( - ) | 0.57 | - |
| Swallow Onset (ms) | 223 | ( 154 ) | 64 | ( - ) | 0.42 | - |

bioRxiv preprint doi: https://doi.org/10.1101/2023.01.18.524513; this version posted January 20, 2023. The copyright holder for this preprint (which was not certified by peer review) is the author/funder, who has granted bioRxiv a license to display the preprint in perpetuity. It is made available under aCC-BY 4.0 International license.

**Table S7.** Provides means, standard deviations (SD), *p*-values and the direction of change for swallow related parameters between male and female mice during water swallows and PiCo stimulated swallow related motor activity in ChAT/Vglut2 mice.

| **Water Swallow** | **Male (*n* =1)** | | **Female (*n* =1)** | | | |
|---|---|---|---|---|---|---|
| | **mean** | **( SD )** | **mean** | **( SD )** | ***p* -value** | **Change** |
| Swallow Duration (ms) | 254 | ( - ) | 218 | ( - ) | - | - |
| XII Duration (ms) | 271 | ( - ) | 236 | ( - ) | - | - |
| Vagus Duration (ms) | 271 | ( - ) | 224 | ( - ) | - | - |
| Laryngeal Complex Duration (ms) | 254 | ( - ) | 257 | ( - ) | - | - |
| *Schluckatmung* Duration (ms) | - | ( - ) | 182 | ( - ) | - | - |
| Diaphragm Inter-Burst Interval (ms) | 736 | ( - ) | 632 | ( - ) | - | - |
| SR Inspiratory Delay (ms) | 457 | ( - ) | 365 | ( - ) | - | - |
| Swallow Sequence (ms) | -42 | ( - ) | 130 | ( - ) | - | - |
| Swallow Onset (ms) | 102 | ( - ) | 73 | ( - ) | - | - |
| **+ ChAT/Vglut2 Stimulation** | **Male (*n* =1)** | | **Female (*n* =2)** | | | |
| **SR Motor Activity** | **mean** | **( SD )** | **mean** | **( SD )** | ***p* -value** | **Change** |
| Swallow Duration (ms) | 171 | ( - ) | 153 | ( 14 ) | 0.48 | - |
| XII Duration (ms) | 173 | ( - ) | 148 | ( 15 ) | 0.39 | - |
| Vagus Duration (ms) | 187 | ( - ) | 154 | ( 6 ) | 0.14 | - |
| Laryngeal Complex Duration (ms) | 195 | ( - ) | 155 | ( 28 ) | 0.45 | - |
| *Schluckatmung* Duration (ms) | - | ( - ) | - | ( - ) | - | - |
| Diaphragm Inter-Burst Interval (ms) | 1023 | ( - ) | 604 | ( 291 ) | 0.45 | - |
| SR Inspiratory Delay (ms) | 507 | ( - ) | 292 | ( 297 ) | 0.66 | - |
| Swallow Sequence (ms) | 22 | ( - ) | -19 | ( 26 ) | 0.42 | - |
| Swallow Onset (ms) | 423 | ( - ) | 240 | ( 49 ) | 0.20 | - |
